# Peer Crowds and Tobacco Product Use in Hawai‘i: A Qualitative Study

**DOI:** 10.3390/ijerph20021029

**Published:** 2023-01-06

**Authors:** Kayzel R. Tabangcura, Rachel Taketa, Crissy T. Kawamoto, Samia Amin, Steve Sussman, Scott K. Okamoto, Pallav Pokhrel

**Affiliations:** 1 Population Sciences in the Pacific Program, University of Hawai‘i Cancer Center, University of Hawai‘i at Mānoa, 701 Ilalo St., Honolulu, HI 96813, USA; 2 Department of Population and Public Health Sciences, Keck School of Medicine, University of Southern California, SSB 302A 2001 N. Soto St., Los Angeles, CA 90033, USA

**Keywords:** young adults, peer crowds, tobacco, e-cigarette, vaping, Native Hawaiian, Asian American

## Abstract

Background: Young adults often derive self-identity from affiliation with peer crowds, which may be defined as reputation-based peer groups centered around characterizable lifestyle norms. Little is known about peer crowds prevalent among Asian American, Native Hawaiian, and other Pacific Islander (AANHPI) populations and the peer crowds’ normative tobacco and other substance use behavior. To address this gap in knowledge, this study conducted focus groups with young adult community college students. Methods: Focus group discussions were conducted with a convenience sample of 42 young adults (Mean age = 21.5, SD = 2.7) recruited across community colleges on O‘ahu, Hawai‘i. The participants represented 60% women, 55% NHPI, and 29% Asian American. Results: Results indicated the presence of a wide range of peer crowds in the population, which may be classified into the following seven categories prevalent in the literature: Regular, Academic, Alternative, Athlete, Geek, High Risk, and Popular. Several peer crowds within the Alternative, Athlete, Geek, High Risk, and Popular categories appeared to represent subcultures relevant for NHPI young adults. High-risk peer crowds were reported to be vulnerable to different types of substance use. Tobacco product use, particularly e-cigarette use or vaping, was noted to be characteristically present among Popular crowds and certain Athlete crowds. Conclusion: Tobacco and other substance use prevention interventions, such as mass media campaigns, may benefit from targeting high-risk peer crowds, especially those relevant for NHPI young adults, who are at high risk for tobacco and other substance use. E-cigarette use prevention interventions may benefit from paying close attention to vulnerable Popular and Athlete groups.

## 1. Introduction

Tobacco-related cancers, such as lung and bronchus cancer, account for the highest proportion of cancer-related mortality in Hawai‘i [1]. Importantly, Native Hawaiian men and women in the state are at markedly higher risks for lung and bronchus cancer incidence and mortality compared with men and women of other ethnic/racial groups. These ethnic/racial disparities in lung and bronchus cancer correspond to the ethnic disparities in tobacco use, especially cigarette smoking. For example, the age-adjusted prevalence of current cigarette smoking among Asian adults in Hawai‘i is approximately 9% compared with 12% among White and 18% among Native Hawaiian and other Pacific Islander (NHPI) adults [2]. The ethnic differences in cigarette smoking are similar across all age groups, especially among young adults [3]. In addition, e-cigarette use prevalence, at 52.4% ever use [4], is higher among NHPI young adults compared with young adults of other ethnic groups [5]. Developmentally, young adults are at an increased risk for transitioning into long-term regular tobacco use [6]. Further, young adults and vulnerable racial/ethnic groups, including AANHPI, have been found to be targeted by tobacco marketing [7,8]. Thus, better understanding the risk and protective factors of tobacco use among young adults, especially NHPI young adults, is of significance.

Informational social influence plays a key role in shaping tobacco product use behavior among young adults [9]. Identity exploration is an integral aspect of young adulthood [10]. As young adults move away from a social environment defined by parents to the one dominated by peers, a need to solidify parameters of one’s self-concept relative to peers becomes increasingly salient [10]. Learning from peers and peer groups helps young adults adopt lifestyle norms and behaviors, which in turn helps them develop a more anchored self and social identity [11].

Peer crowds are reputation-based peer groups that are defined by members’ shared beliefs, behaviors, values, and lifestyles [12]. Peer crowds tend to represent different subcultures and are distinguished by characterizing labels or crowd names such as “Jocks”, “Geeks”, “Skaters”, “Surfers”, and “Hipsters” [13]. Since peer crowds embody prescribed sets of lifestyle norms, peer crowd affiliation facilitates lifestyle-related decision-making for young adults and makes it easier for them to navigate their increasingly complex social environment [9]. Even though peer crowd members may or may not have direct interactions with each other (as a reputation-based collective), they actively engage with the lifestyle and behavioral norms of the peer crowds [13]. These norms may also involve engaging in health risk behaviors such as substance use, risky sexual behavior, and aggressive behavior [13]. For example, affiliation with certain high-risk peer crowds such as “Druggies”, “Gangstas”, and “Hip Hop” has been consistently linked with increased substance use among young people [13]. However, the associations between peer crowd affiliation and substance use across different types of substance use are not always clear-cut.

For instance, past research among adolescents has shown that individuals who self-identify as “Popular” or “Social” [14], or are considered “Popular” or “Social” based on sociometric popularity [15], may be at increased risk for tobacco and alcohol use [14,16]. Young adults who identify as “Country” may be at increased risk for smokeless tobacco use, whereas “Hip Hop” young adults may be more likely to use little cigars [16]. Similarly, young adults who affiliate with athletically oriented groups (e.g., “Gym Rats”) may be more likely to use e-cigarettes but not combustible cigarettes [17]. Conversely, though, young and middle-age adults that frequent vape shops may be more likely to identify with relatively conservative groups [18]. It is possible that peer crowds associated with tobacco use may vary as a function of location (e.g., bar patrons versus vape shops; perhaps geographical regions, East Coast versus West Coast U.S.), and, as in the context of the present study, ethnicity.

### The Present Study

Currently, very little is known regarding young adult peer crowds among Asian Americans, Native Hawaiians, and other Pacific Islanders (AANHPI), especially in relation to tobacco product use. The population of the state of Hawai‘i is predominantly AANHPI. Hawai‘i is geographically isolated from the mainland U.S. and represents a unique culture shaped by Hawaiian history and traditions, as well as by the histories and traditions of the groups that have more recently populated the state’s demography. Yet, Hawai‘i is also part of the U.S., and young adults in the state are also attuned to the mainstream U.S. culture [19]. Hence, the peer crowds prevalent among predominantly AANHPI young adults in Hawai‘i are likely to represent crowds that are unique to the islands, to AANHPI, as well as those that are common in the mainstream U.S. culture. With this understanding, the present study attempted to identify different types of peer crowds that are prevalent among Hawai‘i young adults and characterize the crowds in terms of the members’ characteristics. More specifically, the study attempted to explore the normative presence of tobacco product use behavior across the peer crowds. To these ends, the study conducted focus group discussions with an ethnically diverse sample of community college students on the island of O‘ahu, where two-thirds of Hawai‘i’s population is concentrated. Previous research [20,21,22] suggests that qualitative methods such as focus groups may provide important information in terms of preliminary identification of prevalent peer crowds and their characterization.

Research on peer crowds as related to tobacco use is important because such effort may help guide tailored prevention programming, especially mass media prevention campaigns [23]. Eventually, media campaigns informed by research on peer crowds may help reduce ethnic and other subgroup disparities in tobacco use, and in its health effects, in the state’s multiethnic, multicultural population.

## 2. Methods

### 2.1. Recruitment and Participants

Participants were recruited from community colleges on O‘ahu, Hawai‘i, through use of flyers, word-of-mouth advertisement, and advertisement through e-mail listservs. Students interested in participating in the study contacted research staff by phone. The research staff determined interested students’ eligibility to participate in the study in terms of age (18-to-29 years old), which was the only inclusion criterion. To oversample Native Hawaiian young adults, who are at increased risk for tobacco use compared with other ethnic/racial groups in Hawai‘i [24], we advertised recruitment through clubs and programs that served Native Hawaiian students. Eligible students who provided informed consent were invited to participate in the focus group discussions. Each participant was provided a $45 gift card following focus group participation. The University’s institutional review board approved this study.

### 2.2. Focus Groups

Seven mixed-gender focus groups were conducted, not organized by race or ethnicity, at community colleges across the island of O‘ahu, with each group including 6–8 participants, an appropriate sample size for a structured discussion [25]. See Table 1 for a breakdown of each focus group by gender and ethnicity. Preliminary data analysis after each focus group discussion suggested that by the sixth group, data were beginning to saturate. Thus, we conducted an additional focus group and confirmed that data were saturated. Each group discussion was moderated by the same moderator (PP), a researcher with experience in conducting focus groups and background in peer crowd research. The moderator was accompanied by a note-taker (CTK). All focus group discussions were audio-recorded.

Focus group discussions were carried out in the following manner: First, peer crowds were described to the participants using visual aids. Next, participants were asked to name peer crowds that they had observed in their social environment among people their age. Participants were then asked to describe these peer crowds in terms of appearance, beliefs and values, and lifestyle norms, with special attention to tobacco and other substance use behaviors. Different types of tobacco products, such as combustible cigarettes, electronic cigarettes, hookah (waterpipe), and smokeless tobacco were described to participants. Relevant probes were used as necessary. Discussions lasted 90 minutes, on average, across focus groups.

### 2.3. Data Analysis

All focus group audio recordings were transcribed verbatim by one research assistant, and the transcriptions were validated against the audio recordings by another research assistant. Next, the data were analyzed independently by two research staff members (KT and RT) who were not involved in transcription or transcription validation, using NVivo (Version 12) [26]. Data were analyzed using the principles of inductive content analysis [27]. Following open coding, research assistants (KT and RT) identified unique peer crowd names and coded contexts related to peer crowd names for characterizing descriptions in terms of members’ appearance, values, lifestyle norms, and tobacco and other substance use behavior.

### 2.4. Synthesis of Peer Crowd Names Categories

Based on previous research [17], we expected over 100 peer crowd names to be generated. Sussman et al. (2007) have shown that although there are several peer crowd names, the names may be classified into a fewer number of general categories and such classification systematizes presentation of results and facilitates meaningful discussions [13]. Thus, we followed Sussman et al.’s framework [13] as a starting point and categorized the peer crowd names into the following seven categories that are developmentally appropriate for young adults and have been found to be valid in a previous sample in Hawai‘i [17]: Academic, Alternative, Athlete, Geek, High Risk, Social/Popular, and Regular.

*Regular* represents young adults who do not identify with clearly delineable crowds. For example, they may identify with their grade level at school, ethnicity, or as just being “regular people”. *Academic* represents crowds that embody norms that signify academic focus, educational attainment, and career goals that depend on higher education. *Alternative* includes crowds that signify alternative (i.e., perceived to be non-mainstream) lifestyles. For example, Alternative crowds may place increased value in spirituality and environmentalism; may intentionally maintain an appearance (e.g., sense of dressing) that is considered unconventional; and may engage in careers or recreational activities that are considered artistic, such as theater, photography, and dancing. *Athlete* represents peer crowds that exalt participation in sports, exercising, and recreational activities that involve heightened physical activity. *Geek* includes peer crowds who are overly interested in technological gadgets (e.g., computers, smartphones) and/or video games, or who enthusiastically follow specific aspects of a popular culture (e.g., comic books, certain music). *High Risk* represents crowds for whom high-risk health behaviors such drug use, risky sex, and violence represent the central aspect of their identity. Finally, *Social/Popular* represent crowds whose members want to be perceived as being popular or fashionable among their peers. Members of the social/popular crowds place great importance in their appearance and would want to look good in a conventional or mainstream way. For example, they may dress in clothes of mainstream brands that are expensive and are perceived to be fashionable. Members of popular crowds take pride in their perceived popularity and elevated social status.

To categorize the unique crowd names generated from the focus group discussion into the seven categories, four young adults (two men and two women, each of whom had responded to a call for volunteers) independently sorted flashcards representing each crowd name into the seven categories. The individual sorters completed the tasks in separate sessions, which were scheduled based on their availability over the course of two weeks. Each category was verbally described to the sorters by the last author. Additionally, the sorters were provided a written description of each category for reference during sorting. Disagreement among sorters was reviewed by research team members, and a peer crowd’s final category designation was determined based on consensus.

## 3. Results

### 3.1. Participant Characteristics

A total of 42 participants were recruited. The sample size was determined based on data saturation. Participants ranged between 18 and 27 years in age (Mean = 21.5, SD = 2.7). Sixty percent of the participants were women. The sample was ethnically diverse: 55% of the participants identified as part or full Native Hawaiian, 24% as Filipino, 5% as East Asian (e.g., Japanese, Chinese), 5% as Hispanic, 5% as Other, and 7% as White.

### 3.2. Peer Group Categories

A total of 114 unique peer group names were elicited from the discussions across the two coders (RT and KRT). The list of peer group names generated by the coders were compared for agreement, which was high (Cohen’s Kappa = 0.75). The limited disagreement was resolved through discussions among the co-authors of the current manuscript. The inter-rate reliability among the four young adults who independently sorted the 114 names into the seven aforementioned categories (i.e., Regular, Academic, Alternative, Athlete, Geek, High Risk, and Popular) was relatively high (Cohen’s Kappa = 0.63). Discrepancies across the sorters were resolved through discussions, and consensus, among co-authors. Of the 114 peer group names, 48 were categorized as *Regular*. The remaining 66 peer group names were categorized as *Academic*, *Alternative*, *Athlete*, *Geek*, *High Risk*, and *Popular*. Table 2 lists all 66 peer crowd names by category. The following sections report findings, by category, concerning main crowd names, related lifestyles and tobacco and other substance use characteristics.

#### 3.2.1. Regular

Forty-eight names were classified as *Regular*. These mostly represented names such as “Normal”, “Normal People”, “Averages”, or “Regular People”. Several of the names represented ethnic groups (e.g., Micronesians, Tongans, White boys), religions (e.g., Christian), life-roles (e.g., military wife, married men, mommies), or localities (e.g., Laie boys, west side, east side, Hau‘ula boys/girls, townies). *Regulars* were commonly described as those who did not stand out. Tobacco and/or other substance use was not noted as being normative for *Regulars.*

**Figure 1:**
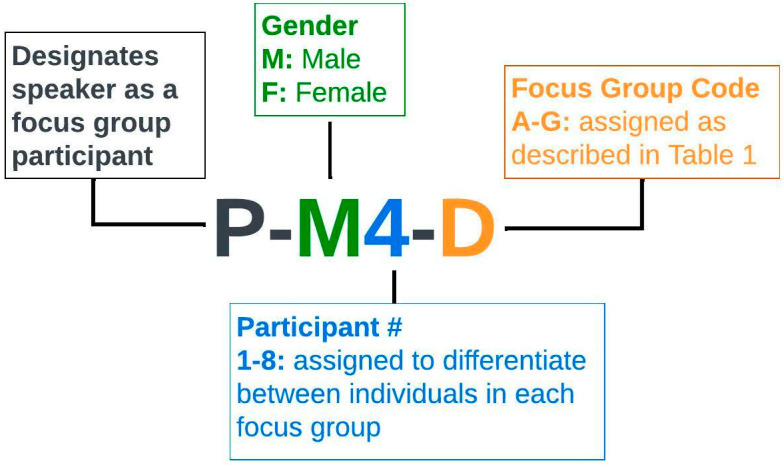
Structure for focus group participant identifier codes in transcripts. # Participant numbers were assigned to differentiate between individuals in each group for the purpose of attributing each transcribed remark to the appropriate individual (e.g., when there are multiple females quoted in a single excerpt).

Figure 1 describes the coding scheme used to differentiate between focus group participants in the transcript excerpts below.

#### 3.2.2. Academic

Three peer crowd names were sorted under *Academic*: Preppy, Scholars, and School People. These groups were described as including individuals who go to school and prioritize academics and scholarly achievements. They are noted to be more studious and are thought to stay away from high risk activities. There were no mentions of tobacco or other substance use in discussions related to these peer crowd names. Participants only described how these peer groups are mostly known for being focused on studies. For example:

“School People”

P-M3-A: *One group I want to add to this is people who go to school because you have people that didn’t go to school and enjoy life. I’m on Instagram and I’ll see someone in Spain again, while I’m getting ready for work. Going to work and school, depending on what you do with your life, that time is one of the hardest things you could go through because you spend 40 hours with school itself, studying, taking tests, and work.*

Moderator: *Is there a name for this kind of people?*

P-M1-A: *School people*.

#### 3.2.3. Alternative

Twelve peer groups were categorized as *Alternative*. Peer groups in the *Alternative* category tended to represent group names such as Woke, Artsy People, Goths, Hippies, Photographers, and Vegans. *Alternatives* were characterized as individuals who were pursuing a way of life that go against the grain of what might be expected. See Table 3 for partial transcripts of focus group discussions of the groups Woke, Goths, and Mālama ‘Āina.

Peer groups in the *Alternative* category represented a variation. They fit the description of Alternative in that they committed to alternative (relative to mainstream or regular) priorities or lifestyles. Yet, they were not primarily described on the basis of deviant behaviors such as violence or substance use, nor academic or athletic proclivities. In addition, their defining lifestyle choices were not circumscribed to a narrow set of activities or hobbies. Tobacco and other substance use was noted particularly in reference to LGBTQ+ individuals:

P-M1-B: *I know for sure in Kalihi we have a huge population of those who like to hang out with those who are in a different sexual orientation. I know that for sure, like LGTBQ, male-to-male, female-to-female. They like to hang out.*

Moderator: *And there’s a subculture surrounding that?...What are their defining characteristics in terms of behavior?*

P-F5-B: *Just thinking about it, they’re into drag culture. They like to hang out at Scarlet [a drag nightclub] in downtown [Honolulu], go to the gay pride events, like the gay parade. They also have gay families. Some of them share a last name that’s not their official last name*.

P-M1-B: *I have family relatives like that, who are LGBTQ, and they smoke more, they do illegal drugs. They’re open because they’re always being judged, so they do stupid stuff.*

P-F5-B: *So you’re saying gay people are more prone to use substances?*

P-M1-B: *From my experience…Some of them are, right out of high school*.

#### 3.2.4. Athletes

The *Athlete* category included six peer groups: Athletes, Football Boys, Gym Rats/Bros, Hikers, Sports Enthusiasts, and Surfers/Spungahs. Members of peer groups in the *Athlete* category were mainly described as individuals who like to engage in sports, activities that involve physical activity, or value physical fitness. Despite the normative emphasis on physical fitness and physical activity, substance use appeared to be part of the lifestyle norms for several of the groups classified as *Athletes*. For example, as transcribed in Table 3, vaping appeared to be common among Gym Bros/Rats, and alcohol and marijuana use among Surfers.

#### 3.2.5. Geek

Seven peer groups were categorized under *Geek*. Peer groups in the *Geek* category represented individuals who were ardent supporters or enthusiasts of certain hobbyist activities or subcultures. Some of the common peer group names included Anime Kids, Koreaboos, K-pop People, Otakus, Weaboos, Gamers, and Nerds. A majority of them were focused on activities such as watching anime or playing video games. Others had hobbies or lifestyle preferences tied to East Asian popular cultures. Participants described the lifestyle and activities of some *Geek* groups, as presented in Table 3. Notably, tobacco product use such as vaping and smoking were noted for some groups such Gamers but not others, like Koreaboos.

#### 3.2.6. High Risk

Seventeen peer groups were classified as *High Risk*. Several peer group names in the *High Risk* category signify some kind of high risk activity that is normative to the groups such as substance use. Some peer groups in the category include Chronics, Kanak Stoners, Ravers, Potheads, and Smokers. These are the peer groups that are more likely to use tobacco products, alcohol, and other drugs. Table 3 provides more specific insights as to High Risk peer groups, as provided by focus group discussion participants.

#### 3.2.7. Popular

Seventeen peer group names were classified under *Popular*. These groups were described as including individuals who put effort into being perceived as popular, glamorous, fashionable, and social. Some of the group names included in the category were: ABGs (Asian Baby Girls), Beauty Gurus, Car People, Hypebeasts, Import Models, and Sneaker Heads, some of which are highlighted in Table 3.

Vaping and other substance use were noted to be common among *Popular* groups:

Moderator: *So vape is very common among hypebeasts? Or sneaker heads? Or ABGs?*

P-M1-C: *Yeah, I think all the subgroups that we’ve told you guys about right now, do vape a lot, so vaping is a really big thing right now*.

## 4. Discussion

Our research set out to identify peer crowds or groups prevalent among predominantly AANHPI young adults from Hawai‘i and describe their normative characteristics, including tobacco and other substance use behavior. Through focus groups with young adults, we identified 114 peer crowd names or social types, which were then grouped successfully into seven broader categories that have been found to be valid among young people in past research [13,17]. This is one of the first studies to have tried to understand young adult peer crowd affiliation in the context of AANHPI and Hawai‘i. Several of the findings may have direct implications for tobacco and other substance use prevention programs and research in the state.

An important finding was identification of *High Risk*, *Alternative,* and *Athlete* peer crowds specific to Hawai‘i that were more likely to engage in tobacco and other substance use. Although a majority of *High Risk* peer crowds that we identified have been widely reported to be prevalent in the mainstream U.S. culture (e.g., Stoners, Ravers) over the years (e.g., Sussman et al. 2007; Nguyen et al. 2020) [13,28], others appear unique to Hawai‘i. For example, the term “Chronic” is specifically used in Hawai‘i to indicate a person addicted to methamphetamine or crack cocaine; elsewhere in the U.S., “chronic”, when used as a slang, refers to a type of marijuana [29]. Similarly, “Moke Bangahz” is specific to Hawai‘i, yet resembles in characteristics the “Gangsta” or “Hip Hop” peer crowds that have been reported in the literature [14,28]. “Kanak Stoner” is another *High Risk* group that has local significance, in that the peer crowd is specific to young Native Hawaiian men who habitually use marijuana. Higher prevalence of marijuana use was also noted for “Spungahs”, an *Athletic* crowd that represents local surfers.

Two local *Alternative* peer crowds that were identified included “Mālama ‘Āina” and “Māhū”. Tobacco and other substance use were not mentioned specifically in reference to “Mālama ‘Āina” and “Māhū”, but tobacco and other substance use was noted to be prevalent among “LGBTQ+”, who were also classified as *Alternative* in the current study. Māhū is a Native Hawaiian term for third gender individuals who have traditionally played the role of spiritual guides in the Native Hawaiian community. More research is needed to better understand whether higher tobacco and other substance use prevalent among the LGBTQ+ peer crowd extends to Māhū as well.

The current findings suggest that *Athlete* groups may be attracted to tobacco products that are commonly perceived to be low in harm, such as e-cigarettes [30]. Upon examining 47,821 responses to the Spring 2017 National College Health Assessment-II Survey, Soule et al. (2021) observed that while college athletes’ use prevalence of combustible cigarettes and waterpipe was lower than that of their non-athlete peers, the same did not apply with regard to e-cigarettes, for which athletes demonstrated comparable or higher usage than non-athletes. The authors speculate that the convenience and lack of evidence linking e-cigarettes with poor athletic performance—relative to cigarette smoking and waterpipe use—contribute to e-cigarettes being perceived as “more compatible with an elite athlete lifestyle” [31].

The current research, which is based on focus groups and others’ report on peer crowds and their characterization, paves way for methodologically more rigorous future studies. Notably, the current findings need further validation through additional qualitative and empirical research. The next first step may be to consolidate the identification of peer crowds though concept mapping or Delphi methods. Additional discussions with ethnically homogenous groups across various AANHPI subgroups may provide further data into ethnic-specific peer crowds. Next, an extensive survey may be conducted to validate the results of the qualitative studies, wherein participants may provide data on self-identification with various peer crowds. If conducted with a representative sample of young adults, such a survey would provide clear indication of the prevalence of various peer crowds and associated normative behaviors, including tobacco and other substance use.

### 4.1. Implications for Drug Prevention and Education

In general, future empirical research needs to better understand the prevalence of tobacco and other substance use in local Hawaiian peer crowds. Importantly, research needs to understand to what extent Native Hawaiian young adults are represented in these groups. Future prevention programs targeted towards Native Hawaiian young adults may benefit from considering the local peer crowds at high risk for tobacco and other substance use. Further, future prevention programs may also need to pay particular attention to some of the *Popular* and *Athlete* crowds that, currently, have been largely missing from the current discourse on peer crowds and tobacco use in the U.S.

The current study delineated several *Popular* crowds such as “Asian Baby Girls (ABGs)”, “Hypebeasts”, and “Car Crews”, that may be at high risk for tobacco product use. These crowds appear to be readily identifiable based on their appearance, clothing, hobbies, and lifestyle preferences. Prevention programs, especially media campaigns, may benefit from tailoring their materials based on the normative features of these groups to directly communicate with members of these peer crowds. In addition, vaping prevention interventions may benefit from paying attention to *Athlete* crowds such as “Gym Bros/Rats”. Due to their tendencies to engage in physical activity or value physical fitness, the prevention needs of *Athlete* peer crowd may not be easily apparent. These groups may benefit from prevention programs that highlight the adverse health effects of vaping.

### 4.2. Limitations

There are several limitations to this study. Since we relied mostly on participants’ reports on their peers’ crowd-identification and related behaviors, the data may be subject to response bias. Information obtained from the participants about the peer groups may reflect stereotypes and preconceived notions, which may in turn have affected the classification of the groups. Participants may also have misrepresented the peer groups that they did not belong to. In addition, the young adult raters who categorized the groups names into the seven peer crowd categories may have had limited knowledge about some peer groups, which, in turn, may have affected group categorization. Moreover, the raters did not completely agree on group categorization. Some groups, such as “Goths”, were classified as *Alternative* by half of the raters, and as *High Risk* by the other half. To classify Goths as *Alternative* involved a subjective judgment on the part of the research team. Thus, we acknowledge that although categorization of peer crowds into broader categories offer a more parsimonious explanation of young people’s social types, and doing so facilitates drawing of practical conclusions, certain peer crowds may not always be categorized into a single higher-order category. Further preliminary analysis involving concept mapping or Delphi methods may help better ascertain different types of peer crowds.

The current study focused on a predominantly Native Hawaiian sample. There may be a need for future studies that examines various AANHPI groups separately. This may provide a better idea of ethnic-specific peer crowds. Lastly, this research was Hawai‘i-specific and conducted among community college students. Some of the peer crowds are rooted in the local Hawai‘i culture. These peer crowds may not be relevant for other parts of the U.S. Additionally, because the sample was drawn from community college students, who tend to overrepresent (compared with four-year colleges) students seeking blue collar professions and students from lower socio-economic status backgrounds [32], there may have been a bias in the study against peer crowd names that are more prevalent in four-year college settings. The same limitation applies in relation to non-college-going young adults. Despite these limitations, however, the current study contributes valuable insight into the nature of peer crowds and their behaviors as related to tobacco and other substance use among young adults in Hawai‘i. The current attempt intended to provide a first look at other-group rated identification among community college-going young adults and also Native Hawaiian young adults sampled through other means, but future work should aim towards tapping larger and representative samples.

## 5. Conclusions

This study corroborated young adult peer crowd affiliations found in the continental U.S. [13]. However, there are a wide range of unique manifestations of broader peer crowd categorizations that are prevalent among young adults in Hawai‘i, and for some of these peer crowds, tobacco and other substance use are normative. Notably, our findings suggest that some of the *High Risk* peer crowds, as implicated by their local names, may represent Native Hawaiian young adults disproportionately more. This, however, needs to be verified through future empirical research. In addition, we found that *Athletes*, despite their higher interest in improving physical fitness and physical activity, may engage in health-risk behaviors such as vaping. This study also highlighted several peer crowds in the *Popular* category that may be at higher risk for vaping and tobacco use. Popular crowds in particular were clearly described by the participants in terms of appearance, clothing, and fashion accessories that are normative to the crowds. Thus, the current study provides important information for tobacco and other substance use prevention programs to consider while tailoring their messages and finding the right medium to communicate the messages to respective peer crowds among young adults in Hawai‘i.

## Figures and Tables

**Table 1:** Overview of Focus Groups.

Focus Group Code Letter	Number of Participants	Participants’ Gender	Participants’ Ethnicity
A	6	3 males, 3 females	4 Filipino, 1 Hispanic, 1 White
B	5	1 male, 4 females	4 Filipino, 1 White
C	4	2 males, 2 females	1 Filipino, 1 Korean, 1 Japanese, 1 White
D	6	2 males, 4 females	4 Native Hawaiian, 1 Filipino, 1 Japanese
E	8	4 males, 4 females	All Native Hawaiian
F	8	4 males, 4 females	7 Native Hawaiian, 1 Hispanic
G	5	2 males, 3 females	3 Native Hawaiian, 1 Tahitian, 1 White

**Table 2:** Peer crowd names by category.

Academic	Alternative	Athletic	Geek	High Risk	Popular
“Preppy” “Scholars” “School people”	“Woke” “Artsy people” “Environmentalist” “Goth” “Hippie” “Hipster” “LGBTQ+” “Māhū” ^1^ “Mālama ‘Āina” ^2^ “Photographer” “Tree hugger” “Vegan”	“Athlete” “Football boy” “Gym rat” “Hiker” “Sports enthusiast” “Surfer” “Spungah” ^3^	“Anime kid” “Koreaboo” ^4^ “Kpop people” ^5^ “Otaku” ^6^ “Weaboo” ^7^ “Gamer” “Nerd”	“Jerks” “Douchebag” “Chronic” “Banger” “Frattie” “Ghetto Kids” “Stoner”/“Kanak Stoner” ^8^ “Loner” “Moke bangah” ^9^ “Narc” “Party animal” “Ratchet” ^10^ “Raver” “Smoker” “Union boy” ^11^ “Wannabe” “Weirdo” “Emo” ^12^ “Ho” “THOT” ^13^	“Beach bum” “ABG” ^14^ “Basic bitch” ^15^ “Beauty guru” “Car people/Car cruisers/Car crew” “Dancer” “Foodie” “Fuck boy” ^16^ “Fuck girl” ^16^ “Hypebeast” ^17^ “IG hoes/model” ^18^ “Import model” ^19^ “Jock” “Pretty boy” “Rich kid” “Sneaker head” “Tita” ^20^

^1^ Māhū: Third gender people with notable religious and cultural roles documented in Native Hawaiian history. In modern culture, more often used as a derogatory term referring to those who identify as LGBTQ+. ^2^ Mālama ‘Āina: Literally “to care for the land”. Used to describe those who are environmentally conscious. ^3^ Spungah: Local people (i.e., not tourists) who enjoy going to the beach for relaxation and/or ocean activities. Sometimes associated with alcohol and marijuana use. ^4^ Koreaboo: A person, typically not of Korean heritage, who is obsessed with Korean culture. ^5^ Kpop people: Fans of Korean pop music. ^6^ Otaku: Anime or manga fanatic. ^7^ Weaboo: A person, typically not of Japanese heritage, who is obsessed with Japanese culture. ^8^ Kanak: Short for “kanaka”, Hawaiian word meaning human. Used colloquially by Native Hawaiians to refer to fellow Native Hawaiians. ^9^ Moke Bangah: Slang term for a Hawai‘i local who likes to “scrap (fight) anybody just for fun”, oftentimes fueled by alcohol. ^10^ Ratchet: A generally crude, obnoxious female who instigates a lot of interpersonal drama. ^11^ Union Boy: Typically refers to men who work in construction and thus belong to trade unions. Wears reflective gear or “highlighter shirts”. ^12^ Emo: Person who is overly emotionally expressive, associated with a rock music genre of the same name that emphasizes emotional expression. ^13^ THOT: Acronym for “That Ho Over There”. Used as a derogatory term to describe a promiscuous woman. ^14^ ABG: Asian Baby Girl, an Asian female usually sporting bleached hair and certain styles of makeup. Enjoys partying. Generally regarded as high-maintenance. ^15^ Basic Bitch: A living stereotype/cliché who enjoys popular mainstream brands. Can be used self-effacingly or as a derogatory depiction of someone else. ^16^ Fuck boy/girl: A promiscuous and self-centered person, regarded by others in the vulgar manner that the term suggests. ^17^ Hypebeast: Person who purchases and wears high-end brands of clothing, shoes, and accessories to appear trendy and establish social status. ^18^ IG hoes/model: Females who dress up to pose for photos they plan to feature on Instagram. ^19^ Import model: Women, typically Asian, who pose for photographs alongside or on top of tuned-up cars at import auto shows. ^20^ Tita: Slang for “sister” in the Hawaiian language. Colloquially, in Hawai‘i, a tita is a no-nonsense local woman who will not back down from a fight.

**Table 3:** Discussion excerpts regarding several peer crowd categories.

*Alternative*	
Peer Crowds	Excerpts
Woke	P-F5-B: Is there a group name for these people that, as you mentioned, like to be involved in the women marches and activism in general? P-F3-B: I think it’s woke. I see it on Instagram. P-F5-B: There’s a hashtag that’s like, “#woke”, like you’re trying to be part of democracy and about civil engagements. Like, you’re awake. P-F3-B: You’re not ignorant to things.
Goths	P-M8-F: Did we get the Goths? Moderator: Tell us more about Goths. P-M8-F: More than likely, they wear dark shades of makeup and piercings all over their body. Listen to rock all the time. Worship Satan maybe. They are just different.
Mālama ‘Āina	P-F4-D: The Mālama ‘Āina group, a lot of them, I feel, they … just like to go outside and cut down trees or whatever, but I feel like a lot of the people who are in the Mālama ‘Āina field do it for their culture, like the Hawaiian culture, and their standards are very cultural-based.
***Athlete***	
**Peer Crowds**	**Excerpts**
Gym Bros/Rats	Moderator: How would you define a gym rat? P-F6-A: Goes to the gym every day, works out at the gym every day. P-M3-A: They don’t have to work, go to gym. P-F5-A: Carry around a protein shake every day. P-M3-A: Meal prep. P-M4-A: Protein, chicken, eggs. P-F5-A: Broccoli. Moderator: Do they vape? P-M2-A: A lot of them … Hawai‘i is like, “I’ll go to the gym but we’re going to have a beer later because we can burn it off tomorrow at the gym”. But smoking-wise, like, “Oh, I have to get my cardio up, but I’m going to vape right after”. I’ve seen that happen so many times. Moderator: So you think gym rats more often would be vaping rather than smoking. P-M2-A: More vaping, yeah. P-F1-A: Vaping is too permanent of a thing. P-M2-A: Yeah, because gym rats are more focused on muscle building rather than cardio, but they do vape. Like, I passed this new gym in Ala Moana, some Planet Fitness, and I saw a guy adding stuff to his vape inside the gym and he’s vaping at the same time, like I think he’s contradicting everything. I just saw that last night, so I was just tripping out.
Spungahs? (*pronounced SPUN-jahz*)	P-F5-D: Spungahs. P-M2-D: It’s in tandem with surfers. P-F5-D: It’s more like local people, though. Surfers can be like foreign people that come here to surf, so down here there’s spungahs. Local people like ourselves that go to the beach a lot and smoke weed. My friends from high school were top spungahs. They would smoke weed, drink, and just have a good time.
***Geek***	
**Peer Crowds**	**Excerpts**
Anime Kid	Moderator: Could you help me visualize an Anime kid? P-F4-F: They have crazy hair colors. I don’t know how to explain them. P-M8-F: Kids that wear stuff that people wouldn’t call normal. They wear things that come out of animated cartoons, like a Pikachu hat, or a Pikachu one-piece. P-M1-F: The cough masks. P-M8-F: They paint their faces. P-M1-F: They cosplay. The Japanese thing, costume play. P-M8-F: But usually they’re not Japanese. P-M1-F: Sometimes they wear claws, mittens, whiskers.
K-pop People	P-F2-F: [K-pop people] have a bunch of things: They have a lot of fan groups, so there’s apps that you can have for that one group that you really like. There’s a whole community, and they set up events, and there’s fans that fly out and meet.
Gamers	P-M2-D: The first group that comes up is gamers. I have to say it because I play games every day. I wouldn’t say that I embody that group, or present it all the time, but I play games every day. Moderator: Is there a behavior in addition to gaming that applies to gamers? P-M6-D: I play games. A lot of them smoke, a lot of my friends who I’ve connected with online, they all tend to smoke. Moderator: These are all gamers? P-M6-D: Yeah. P-F5-D: Smoke cigarettes? P-M6-D: I know a lot of gamers, and there’s definitely those who smoke to get high and play games at the same time. I know those who take breaks after intermissions of MPG, they’ll be like, “I’m going to smoke real quick, be right back”. P-M2-D: There’s a lot of YouTubers or Twitch-streamers, people that stream themselves playing games. Twitch is a website where you can stream yourself playing games and make money. In that, you can see some people smoke while they play, they swear, whatever. They drink, some people make a game out of it, and they’ll drink while playing. Moderator: Do they vape as well? P-M2-D: I would say more recently, I’ve seen a lot more electronic [cigarettes]. P-M6-D: Because they can do it inside their room or certain places. Moderator: What about alcohol use among gamers? Is it common? P-M2-D: I can’t say for sure. I don’t want to be drunk and play games. P-M6-D: I love it! I can just talk to people when I’m, you know, and get competitive. But I know there’s certain groups that all drink and play games, and they all just have a good time. Moderator: These are gamers too? P-M6-D: Yeah.
Koreaboo	Moderator: So these groups that are obsessed with Japanese and Korean cultures, is smoking or vaping more prevalent in the groups or no? P-F5-A: A lot of them don’t, only because it’s like, “Oh, we’re introverted, we just want to stay inside and stay up until 2 in the morning to watch our groups perform” and stuff like that, from what I’ve seen. But I do know that there’s a lot of them that were past smokers, and they’re like, “Oh, this is my new outlet now”. So I’ve had maybe 4 or 5 friends like that.
***High Risk***	
**Peer Crowds**	**Excerpts**
Chronics	Moderator: Could you describe chronics? P-M2-E: You can just tell, you have to be around to just know. P-M3-E: Some chronics are crazy. They think people are talking to them, or they tweak out and think someone’s trying to rob them. P-M4-E: I used to work with this guy, you wouldn’t tell, but when he would work, he would clean everything, like full on. I used to work at a kitchen; pull out all the refrigerators, scrub the floor, under the refrigerators, clean out the refrigerators, like just yeah. P-M2-E: Chronics our age, they wouldn’t be here, in school. P-M3-E: They’d probably be chilling on the block somewhere.
Stoners and Ghetto Kids	P-F4-F: What I see are… The ghetto kids in Wai‘anae, like literally ghetto. Some of them don’t brush their teeth, don’t wear slippers … Then there’re the stonies, and they just chill, mind their own business, take care of themselves, smoke weed. Moderator: The names that you’ve mentioned, is tobacco use, e-cig use, common among some more than others? P-M8-F: More than likely those, yeah. Moderator: Who’s it common among? P-F4-F: The stonies and the ghetto kids.
Moke Bangahz	P-M2-E: Get the moke bangahz, da kine bonfire, but every day bonfire, party, fish, dive, scrap anybody just for fun, drink beer, you know. Swear every f-word, and it’s not even negative, that’s just part of their vocabulary. Moderator: These are the moke bangahz? P-M2-E: Yeah, and if you want to get into what they wear … Moderator: Go ahead. P-M2-E: Okay, there’s levels to this moke bangahz, there’s the moke braddahs that wear slippers, surf shorts, t-shirt … And they get the one that dress to kill, moke bangahz, who get the [gold] chain, the nice shirt, the jeans. I’ll bark at them, but they’ll bark back.
Party Animals	P-F8-E: Party animals party a lot. P-M2-E: They start drinking, they want to smoke. P-M3-E: We got those drinking, smoking. P-F8-E: Cross-faded. P-M3-E: They only smoke cigarette when they drink, but they don’t want to buy their own pack.
Ravers	P-M1-C: Ecstasy for the ravers. P-M3-C: For ravers, ecstasy is one of the top ones. Moderator: Is vaping culture big among ravers too? P-M1-C: Absolutely. Moderator: And they vape stuff other than nicotine? Do you know? P-F4-C: I think it’s more common, but I’ve heard of some people who use it for marijuana stuff. I forgot what it’s called. P-M3-C: Dab? P-F4-C: Yeah. P-M3-C: They have the THC dab.
***Popular***	
**Peer Crowds**	**Excerpts**
ABGs	P-F4-C: Not to offend anybody, but I think ABGs are considered more attractive? Or the popular … Yeah. Moderator: Can you describe ABGs a bit more? P-F2-C: I know some people. Most of them are from the west side, a group of girls, and they’re really into eyelashes, makeup, like going all out … Spending two hours on their makeup. When they go out, they wear bling everything. They wear boots covered over their knees. You would think you wouldn’t dress like that in Hawai‘i. I do not know what you would call them … Boujee?
Hypebeasts	P-F4-C: Hypebeasts. P-M1-C: Hypebeasts are more like clothing too. Street style. P-F4-C: Guys who are really into hats and shoes, but it’s more like a specific type of clothing that they’re into. P-F2-C: Like Supreme.
Car People	P-F1-A: The car people, if you look at them, you know they’re a car or motorcycle person. Moderator: Why? P-F1-A and P-F5-A: The way they dress. Moderator: How do they dress? P-F1-A: Expensive wear. P-F5-A: They wear a lot of street wear. My boyfriend is part of a car crew and his group, all of them are sneakerhead, like that’s their thing, and if they do vape, they like to do it out in open parking lots. They never do it in their car because they don’t want their car to smell like the vape.

# Pre-assigned participant numbers designate individuals within each group for the purpose of attributing each transcribed remark to the appropriate individual. For example, in this excerpt regarding the Woke peer group, two females (F5 and F3, respectively) Focus Group B are speaking in turn.

## Data Availability

Not applicable.
